# Minimal Effects of Medium-Chain Triglyceride Supplementation on the Intestinal Microbiome Composition of Premature Infants: A Single-Center Pilot Study

**DOI:** 10.3390/nu14102159

**Published:** 2022-05-22

**Authors:** Jesús A. Romo, Amanda B. Arsenault, Sonia S. Laforce-Nesbitt, Joseph M. Bliss, Carol A. Kumamoto

**Affiliations:** 1 Department of Molecular Biology and Microbiology, Tufts University School of Medicine, 136 Harrison Ave., Boston, MA 02111, USA; jesus.romo@tufts.edu; 2 Department of Pediatrics, Women & Infants Hospital of Rhode Island, Providence, RI 02905, USA; aarsenault@geisinger.edu (A.B.A.); slaforcenesbitt@wihri.org (S.S.L.-N.); jbliss@wihri.org (J.M.B.); 3 Warren Alpert Medical School, Brown University, Providence, RI 02903, USA

**Keywords:** premature infants, microbiome, medium-chain triglyceride, Enterobacteriaceae, supplementation

## Abstract

Compared to term infants, the microbiota of preterm infants is less diverse and often enriched for potential pathogens (e.g., members of the family Enterobacteriaceae). Additionally, antibiotics are frequently given to preterm infants, further destabilizing the microbiota and increasing the risk of fungal infections. In a previous communication, our group showed that supplementation of the premature infant diet with medium-chain triglyceride (MCT) oil reduced the fungal burden of *Candida* spp. in the gastrointestinal tract. The objective of this study was to determine whether MCT supplementation impacts the bacterial component of the microbiome. Pre-term infants (*n* = 17) receiving enteral feedings of either infant formula (*n* = 12) or human milk (*n* = 5) were randomized to MCT supplementation (*n* = 9) or no supplementation (*n* = 8). Fecal samples were taken at randomization and prior to MCT supplementation (Week 0), on days 5–7 (Week 1) and day 21 (Week 3). After DNA extraction from samples, the QIIME2 pipeline was utilized to measure community diversity and composition (genera and phyla). Our findings show that MCT supplementation did not significantly alter microbiota diversity or composition in the gastrointestinal tract. Importantly, there were no significant changes in the family Enterobacteriaceae, suggesting that MCT supplementation did not enrich for potential pathogens. MCT holds promise as a therapeutic intervention for reducing fungal colonization without significant impact on the bacterial composition of the host gastrointestinal tract.

## 1. Introduction

The human microbiota is acquired early in life via transmission from mother to child during birth (maternal vaginal and fecal bacteria [1,2,3]) and from the environment encountered during delivery [4]. The infant microbiome undergoes rapid changes throughout the first two years of life [5] on the path to maturation and stabilization. The microbiome is impacted by a variety of factors, including route of delivery [3], gestational age [6,7], human milk vs. formula diet [1,8,9], and the environment (e.g., the presence of siblings) [10], among others. Importantly, the assembly of the microbiota at this early stage impacts long-term health [11,12,13,14]. The microbiota assembly, maturation, and composition in healthy infants, along with the factors that influence it, have been well-characterized by large longitudinal studies, such as the Canadian Healthy Infant Longitudinal Development (CHILD) and The Environmental Determinants of Diabetes in the Young (TEDDY). Data from these studies have been used to further understand the role of the infant microbiota in the development of asthma [15,16] and allergies [17]. Findings from studies such as these underscore the importance of bacterial genera, such as *Faecalibacterium*, *Lachnospira*, *Veillonella*, and *Rothia* (FLVR), during the first 3 months of life, as infants with low levels or lacking FLVR bacteria are more prone to developing asthma [15]. These studies have yielded a wealth of information on the development of the infant microbiome early in life and the role it plays in health and disease, but there is still a relative lack of information regarding the microbiota composition in premature infants.

Premature infants have an immature gastrointestinal tract with low epithelial barrier function and high permeability, allowing for the translocation of bacteria from the gut to the bloodstream, which can cause systemic inflammation or sepsis [18,19,20,21,22]. The premature infant microbiota has been characterized as less diverse and lacking taxa found in healthy infant guts. These healthy taxa have been associated with training the immune system, as well as being important for decreasing the risk of obesity, allergies, and asthma [15,17,23,24,25]. The premature microbiota is also known to be enriched with pathogenic bacteria, which have been linked to systemic inflammation, sepsis, and necrotizing enterocolitis (NEC) [26,27,28]. To combat these infections, premature infants have frequent exposure to broad-spectrum antibiotics. Antibiotic treatment has been shown to significantly increase susceptibility to fungal infections in infants [29].

Importantly, most infant microbiota studies do not consider the mycobiome, which is the fungal component of the microbiome. These organisms make up an estimated 0.1% of the microbiota in the healthy adult GI tract [30], yet their impact on health and disease is poorly understood. Premature infants have an increased risk for severe invasive infections caused by fungi from the genus *Candida,* including *C. albicans,* which is a colonizer of the gastrointestinal tract acquired early in life [31,32]. To reduce the risk of fungal infections, antifungals are often used prophylactically in premature infants [33]. However, studies have shown that once *Candida* colonizes the gastrointestinal tract, antifungal prophylaxis is less effective [34]. Previously, our group demonstrated that dietary medium-chain fatty acids significantly impacted *C. albicans* gastrointestinal colonization in a murine model [35]. A subsequent study in human preterm infants demonstrated that dietary supplementation with medium-chain triglyceride (MCT) oil, a commonly used component of infant formula, significantly reduced gastrointestinal *Candida* colonization. These findings suggest that MCT oil administration could be a safer treatment for the reduction of *Candida* colonization in the already fragile microbiome of premature infants [36]. Here, we investigate the impact of MCT oil supplementation on the bacterial component of the microbiota of premature infants. We further separate the MCT and control infants based on feeding practices (human milk vs. formula).

## 2. Materials and Methods

### 2.1. Study Participants and Eligibility

The fecal samples used in this study were collected as part of a pilot trial conducted at the Women & Infants Hospital of Rhode Island, as described previously [36]. Briefly, premature infants admitted to the NICU were screened for gastrointestinal colonization by *Candida* between June 2014 and December 2015 and from January 2019 to February 2020. Infants born prior to 37 weeks’ gestation who had attained full enteral feedings with either fortified human milk or commercial preterm infant formula (Similac Special Care, Abbott Nutrition, Lake Forest, IL, USA, or Enfamil Premature, Mead Johnson Nutrition, Evansville, IN, USA) were eligible for screening. Bovine-based fortifiers were used to fortify human milk. Infants were excluded from the study if their gestational age was greater than 37 weeks or if they were currently receiving parenteral nutrition or MCT oil supplementation, were exposed to antifungals, presented with GI anomalies, or were expected to stay in the hospital for less than three weeks.

### 2.2. Sample Collection

A stool sample was cultured from all eligible infants, and the parents of those with detectable yeast were approached for informed consent. Daily stool samples were collected from consenting infants for 3 days prior to randomization, and then infants were randomized in an unblinded fashion to receive supplemental dietary MCT oil (Nestlé Health Science, Vevey, Switzerland) at a dose of 0.5 mL/oz (4 kcal/oz), or to receive no supplementation for all feeds over 7 days. Following publication of the initial study, the protocol was modified to continue supplementation for 21 days and stool samples were collected as close as possible to day 7 and day 21. The samples taken at randomization and prior to MCT supplementation (Week 0), on days 5–7 (Week 1) and day 21 (Week 3; infants studied for 21 days only) were used for this study. Importantly, the number of samples collected on Week 3 decreased from 17 to 6. For unknown reasons, between 2016 and 2019 there was a decrease in the frequency of infants with fungal colonization, limiting the number of participants we were able to recruit for the longer study in 2019 and 2020. Stool samples were weighed, suspended in 1 mL of sterile 0.9% NaCl, and promptly frozen at −80 °C for later microbiota analysis.

### 2.3. DNA Extraction, 16s rRNA Amplification, Library Preparation, and Sequencing

Microbial DNA was extracted using the QIaAMP PowerFecal Pro DNA Kit (Qiagen, Hilden, Germany, cat. 12830-50) according to the manufacturer’s protocols. Libraries were prepared from each sample and sequenced as previously described [37]. Briefly, PCR amplification of the V4 region of the 16S rRNA gene was performed with 515F and 806R primers that included adapters for Illumina sequencing and 12-mer Golay barcodes to allow for multiplexing. Two hundred and fifty bp paired-end sequencing was performed using an Illumina MiSeq according to the manufacturer’s protocols.

### 2.4. Microbiota Analysis

Base calling was conducted with CASAVA 1.8 and the resulting fastq files were used for downstream analysis using QIIME 2 (2018.8) [37]. Demultiplexing and filtering of raw sequences was conducted using the q2-demux plugin, followed by DADA2 [38] for denoising (q2-dada2). Amplicon sequences were aligned using mafft [39] (q2-alignment). Phylogeny trees were constructed using the de novo phylogenetic tree from fasttree2 [40] (q2-phylogeny). Alpha-diversity metrics (Shannon, Chao1, Simpson, and Faith’s PD) and beta diversity (weighted UniFrac [41]) were estimated using the q2-diversity. Principal coordinate analysis (PCoA) was used to summarize the weighted UniFrac distance matrix. PERMANOVA analysis was performed using QIIME2. The operational taxonomic units (OTUs) were determined by aligning reads to the Greengenes Database (version 13_8) at 99% identity [42]. Analysis of compositions of microbiomes (ANCOM) [43] was used to compare relative abundance of bacterial taxa within each sample.

### 2.5. Statistical Analyses

Statistical analyses were performed using QIIME2 (PERMANOVA and ANCOM) and GraphPad Prism (San Diego, CA, USA). *p* < 0.05 was used as the cutoff for statistical significance.

## 3. Results

### 3.1. Pre-Term Infants Harbor a Limited Gut Microbiota Composition Dominated by Enterobacteriaceae and Not Impacted by MCT Supplementation

The first years of an infant’s life represent a highly dynamic time for the microbiome as it develops and transitions to a stable community [9,44]. For preterm infants, these microbial communities are typically less diverse than those of full-term infants [6,24,25,27,45,46]. To characterize the microbiota composition of preterm infants in this study, the 16S rRNA gene amplicons obtained from fecal samples acquired from control infants (*n* = 8) and MCT fed infants (*n* = 9) collected before treatment, and one or three weeks after daily supplementation were analyzed. Demographic and clinical characteristics of the study population are summarized in Table 1.

**Figure 1:**
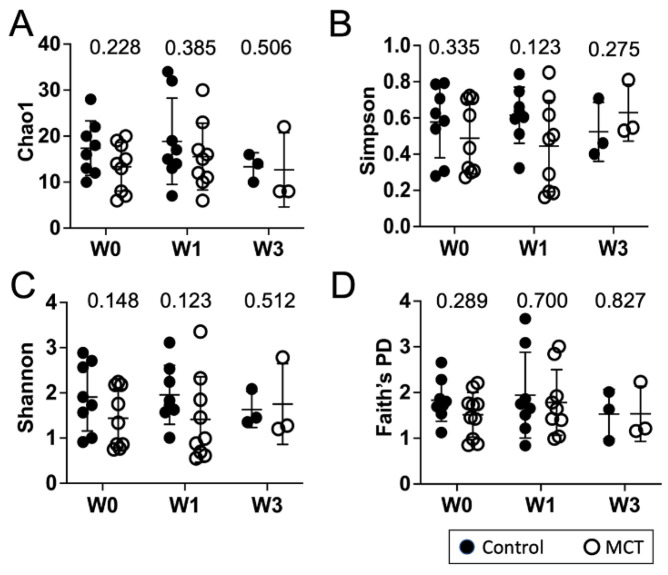
Infant gut microbiota diversity is not impacted by MCT supplementation. Stool samples were collected from infants before (Week 0), or after 1 or 3 weeks of supplementation with MCT. Control infants were sampled at the same times but did not receive MCT. Bacterial composition of control and MCT-supplemented infants over three weeks was analyzed by 16S rDNA sequencing, followed by QIIME2 analysis. Diversity scores were calculated. The bar represents the mean diversity score with the standard deviation, for all infants within a group. (**A**) Chao1, (**B**) Simpson, (**C**) Shannon, and (**D**) Faith’s PD. Black circles denote control infants. Open circles denote MCT-supplemented infants. Mann–Whitney *p*-values are displayed above each set.

**Figure 2:**
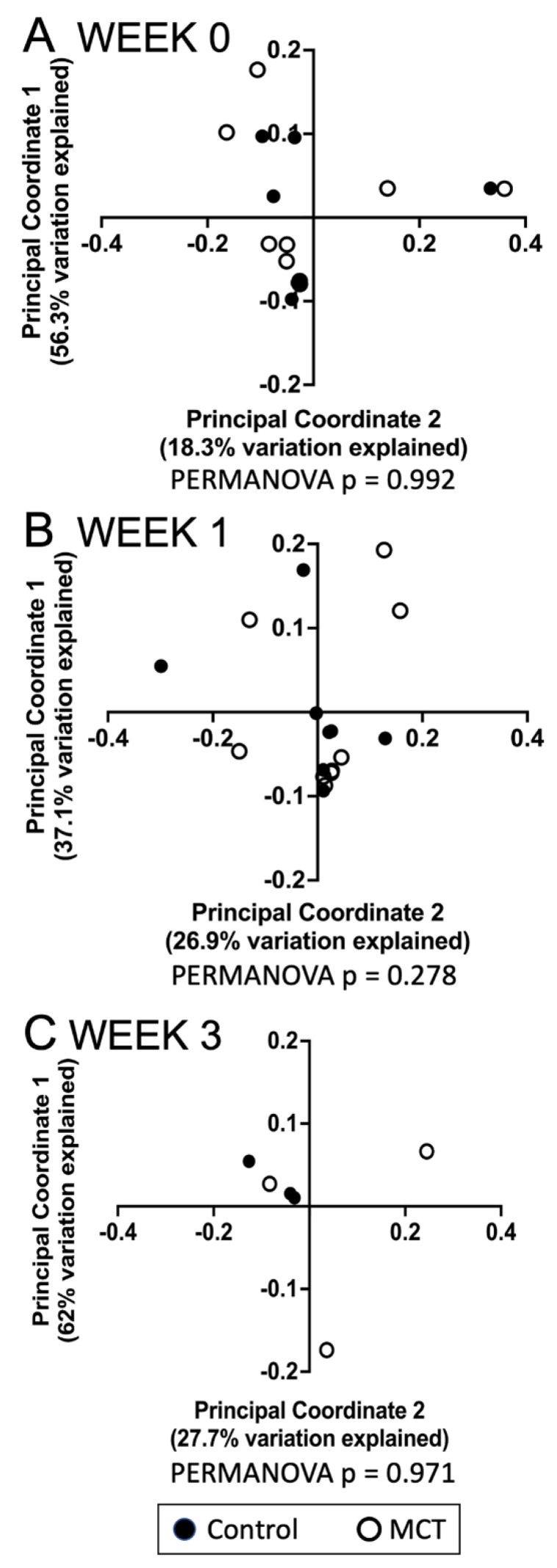
Analyses of gastrointestinal bacterial communities in control or MCT-supplemented infants. Beta diversity of bacterial communities in stools from control and MCT-supplemented infants over three weeks was analyzed using QIIME 2. Weighted UniFrac distances were used to perform a principal coordinate analysis between groups at different time points. (**A**) Week 0 indicates samples collected before supplementation began. (**B**) Week 1 indicates samples collected after one week of MCT supplementation. (**C**) Week 3 indicates samples collected after three weeks of MCT supplementation. Black circles denote control infants. Open circles denote MCT-supplemented infants. PERMANOVA *p*-values are displayed below each graph.

To identify a possible effect of MCT supplementation on community diversity, community diversity was measured using Chao1, Simpson index, Shannon index, and Faith’s PD (Figure 1A–D). Alpha diversity analyses showed no significant temporal changes between treated and untreated groups. Moreover, the gut microbiota composition was compared between groups using the weighted UniFrac distance metric for multivariate analyses. The principal coordinate analysis (PCoA) of the weighted UniFrac distance for the bacterial communities in the control versus the MCT infants is shown in Figure 2. PERMANOVA analysis did not detect statistically significant differences between the control and MCT group at any timepoint (pre-treatment *p* = 0.992; Week 1 *p* = 0.278; Week 3 *p* = 0.971). These findings demonstrate that MCT supplementation did not have significant effects on bacterial microbiota diversity.

**Figure 3:**
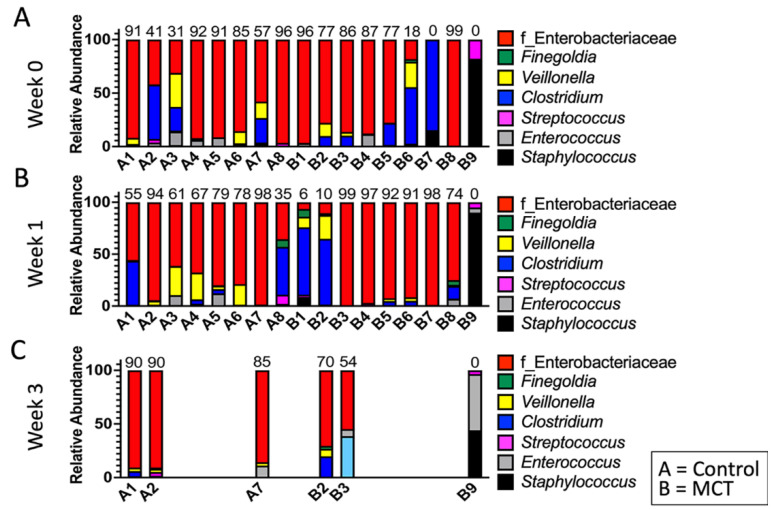
Infant gut microbiota is dominated by Enterobacteriaceae and is not impacted by MCT supplementation. Top bacterial taxa observed in stool bacterial communities are displayed over three weeks. (**A**) Week 0 indicates samples collected before supplementation began. (**B**) Week 1 indicates samples collected after one week of MCT supplementation. (**C**) Week 3 indicates samples collected after three weeks of MCT supplementation. Infants A1–A8 belong to the control group. Infants B1–B9 belong to the group that received MCT. Only six infants remained in the study by week 3. Numbers above bars represent the percentage of Enterobacteriaceae in a particular infant.

**Figure 4:**
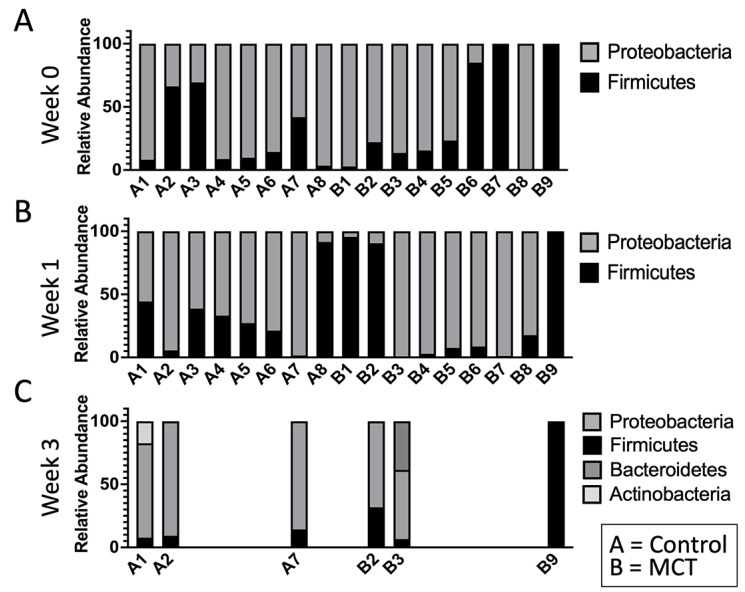
The infant gut microbiota is composed of limited phyla. Phyla were observed in stool bacterial communities over three weeks. (**A**) Week 0 indicates samples collected before supplementation began. (**B**) Week 1 indicates samples collected after one week of MCT supplementation. (**C**) Week 3 indicates samples collected after three weeks of MCT supplementation. Infants A1–A8 belong to the control group. Infants B1–B9 belong to the group that received MCT. Only six infants remained in the study by week 3.

To test for possible differences in bacterial genera between control and MCT-treated infants, the relative abundance of bacterial genera was compared. All bacterial genera with a median greater than 0 in at least one of the groups were included in the analysis (Figure 3). As described above, the microbiota of preterm infants lacks diversity, and these samples contained few genera. Interestingly, the only member of FLVR represented was Veillonella (Figure 3). Furthermore, the microbiota of most infants was dominated by members of the family Enterobacteriaceae (Figure 3). This relative abundance of Enterobacteriaceae persisted throughout the study, and has been observed in previous studies with preterm infants [7,46,47,48], further highlighting the high degree of dysbiosis in preterm microbiota. Importantly, the relative abundance of Enterobacteriaceae did not show any patterns and changed similarly in both groups, suggesting that MCT supplementation did not lead to higher relative abundance of Enterobacteriaceae. Additionally, we did not detect any significant differences in phylum composition between the two groups at any timepoint (Figure 4).

These findings suggest that MCT supplementation did not have a significant impact on host microbiota composition and did not increase the relative abundance of members from the Enterobacteriaceae family, which have been associated with an increased risk of necrotizing enterocolitis (NEC) [26,27,28].

### 3.2. Human Milk vs. Formula Feeding

Previous studies have highlighted the significant impact that diet, and specifically human milk, can have on the infant microbiome [1,9,49,50,51]. Some infants in our study exclusively received fortified human milk (n = 5) while others received preterm infant formula (n = 12). We tested for changes in microbiota composition due to a combination of type of feeding and MCT supplementation. Measurements of community diversity (Chao1, Simpson index, Shannon index, and Faith’s PD) detected a statistically significant difference between control and MCT-treated infants, who were fed a preterm formula diet with one metric, Simpson index (Supplemental Figure S1F). These findings suggest that MCT-treated infants had a less diverse microbiome compared to control when given a preterm formula diet. Importantly, the remaining alpha diversity analyses showed no significant differences between the diets and MCT treatment (Supplemental Figure S1).

The principal coordinate analysis (PCoA) of the weighted UniFrac distance for the bacterial communities in the control versus the MCT infants given different diets (fortified human milk vs. preterm formula) is shown in Supplemental Figure S2**.** PERMANOVA analysis did not detect statistically significant differences between the groups at any timepoint or with any diet (pre-treatment *p* = 0.633; Week 1 *p* = 0.09; Week 3 *p* = 0.13). Taken together, these findings demonstrate that MCT supplementation did not have a substantial effect on bacterial microbiota diversity, regardless of diet.

**Figure 5:**
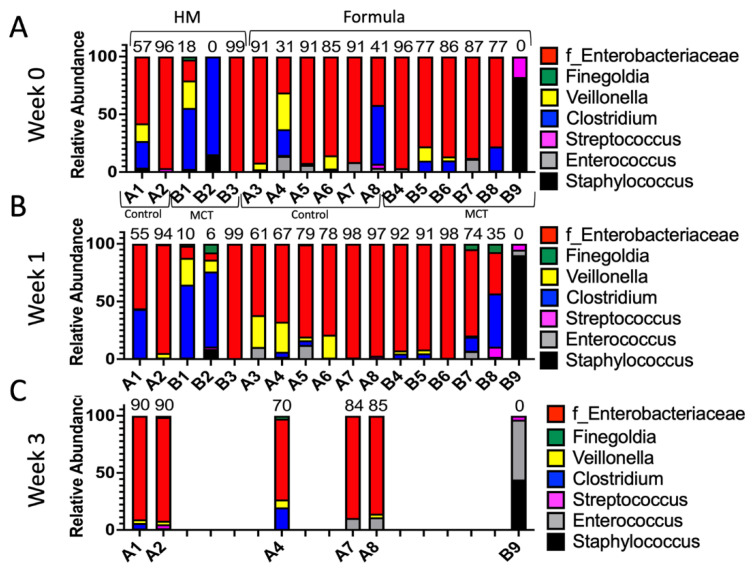
Infant gut microbiota of control vs. MCT-supplemented infants under a human milk or formula diet. Infants were separated into groups based on their type of feed and their stool bacterial communities, as displayed. (**A**) Week 0 indicates samples collected before supplementation began. (**B**) Week 1 indicates samples collected after one week of MCT supplementation. (**C**) Week 3 indicates samples collected after three weeks of MCT supplementation. Infants A1–B3 belong to the human milk group. Infants A3–B9 belong to the formula group. Only six infants remained in the study by week 3. Numbers above bars represent the percentage of Enterobacteriaceae in a particular infant at week 3.

To test for possible differences in bacterial genera between control and MCT-treated infants fed different diets, we compared the relative abundance of bacterial genera. As expected based on the findings described above, no significant differences in relative abundance of phyla or genera were detected between the groups (control vs. MCT) or between different diets at any timepoint (Figure 5 and Supplemental Figure S3).

Overall, these findings indicate that MCT supplementation exerted minimal impacts on the overall diversity of the microbial community.

## 4. Discussion

The infant microbiota composition plays a significant role in the health of the individual well into adulthood. The preterm infant microbiota has been characterized as being less diverse and harboring potential pathogens, such as those associated with an increased risk of NEC. Additionally, antibiotics are commonly prescribed, further disrupting the microbiota and leading to a predisposition for fungal infections. Previously, our group evaluated the effect of dietary MCT supplementation on colonization with the fungus *Candida* in preterm infants [36]. MCT significantly reduced *Candida* colonization in the gastrointestinal tract, suggesting the potential utility of MCT oil as a dietary prophylactic. Here, we characterized the impact of MCT supplementation on the microbiota composition of the same preterm infants. As expected, the preterm microbiota showed a trend towards dysbiosis, as evidenced by the lack of diversity and dominance by Enterobacteriaceae. Although limited by the small sample size (17 infants total), our findings suggest that MCT supplementation had no impact on community diversity, except in the subgroup of infants fed formula diets. Importantly, MCT treatment did not lead to increased relative abundance of Enterobacteriaceae, which underscores its potential as a safe dietary intervention to prevent fungal infections.

It is important to acknowledge that this pilot study holds several limitations, including a small sample size (17 infants), short supplementation time, and variations in the ages of infants, all of which could impact microbiota composition. Moreover, the number of infants recruited for the 3-week study was low, because the rates of fungal colonization were low for infants studied in 2019 and 2020. This low colonization rate provides important information for the design of a full clinical trial of MCT supplementation in preterm infants. To achieve an adequate sample size, a multi-centered approach would likely be needed. Finally, the analysis relied on measurements of microbiota composition, and absolute abundances of taxa were unknown.

Even with these caveats, our findings are useful as they highlight the potential for MCT as a dietary supplement to lower *Candida* colonization within a short period of time without having significant impacts on the infant microbiome. These findings support the need for larger studies to further characterize the utility of MCT in the clinical setting.

## 5. Conclusions

In summary, our group previously identified a dietary intervention that successfully reduces numbers of fungal colonizers in the gastrointestinal tract of infants [36]. Here, we further the study by showing that this dietary intervention does not significantly impact the composition of the bacterial component of the microbiome. MCT supplementation could thus represent a safer and less disruptive approach for reducing fungal colonization in infants at high risk for fungal infection.

## Figures and Tables

**Table 1:** Demographics.

	Control (*n* = 8)	MCT (*n* = 9)
Maternal age, years *	29 (18–36)	27 (19–38)
Male gender, *n* (%)	4 (50)	6 (67)
Gestational age, weeks *	28.9 (25–33)	27.2 (23–32)
Cesarean delivery, *n* (%)	5 (63)	6 (67)
Any prenatal antibiotic, *n* (%)	5 (63)	8 (89)
Any prenatal steroid, *n* (%)	5 (63)	8 (89)
Birth weight, g *	1229 (500–2010)	876 (360–1220)
Any breast milk, *n* (%)	7 (88)	7 (78)
Exclusive breast milk, *n* (%)	1 (13)	3 (33)
Parenteral nutrition days *	10 (4–26)	18 (4–53)
Age at enrollment, days *	27 (13–55)	34 (9–90)

* mean (range).

## Data Availability

The raw sequencing data were submitted in FASTQ format to the NCBI Sequence Read Archive. The BioProject ID is PRJNA813890.

## Supplementary Materials

The following supporting information can be downloaded at: https://www.mdpi.com/article/10.3390/nu14102159/s1, Figure S1: Infant gut microbiota diversity is moderately impacted by MCT in infants fed a formula diet; Figure S2: Analyses of gastrointestinal bacterial communities in control or MCT-supplemented infants who received a human milk or formula diet; Figure S3: Gut bacterial phyla from infant stool, grouped by diet.

## References

[1] Backhed F., Roswall J., Peng Y., Feng Q., Jia H., Kovatcheva-Datchary P., Li Y., Xia Y., Xie H., Zhong H. (2015). Dynamics and Stabilization of the Human Gut Microbiome during the First Year of Life. Cell Host Microbe.

[2] Dominguez-Bello M.G., Costello E.K., Contreras M., Magris M., Hidalgo G., Fierer N., Knight R. (2010). Delivery mode shapes the acquisition and structure of the initial microbiota across multiple body habitats in newborns. Proc. Natl. Acad. Sci. USA.

[3] Shao Y., Forster S.C., Tsaliki E., Vervier K., Strang A., Simpson N., Kumar N., Stares M.D., Rodger A., Brocklehurst P. (2019). Stunted microbiota and opportunistic pathogen colonization in caesarean-section birth. Nature.

[4] Ferretti P., Pasolli E., Tett A., Asnicar F., Gorfer V., Fedi S., Armanini F., Truong D.T., Manara S., Zolfo M. (2018). Mother-to-Infant Microbial Transmission from Different Body Sites Shapes the Developing Infant Gut Microbiome. Cell Host Microbe.

[5] Charbonneau M.R., Blanton L.V., DiGiulio D.B., Relman D.A., Lebrilla C.B., Mills D.A., Gordon J.I. (2016). A microbial perspective of human developmental biology. Nature.

[6] Brooks B., Firek B.A., Miller C.S., Sharon I., Thomas B.C., Baker R., Morowitz M.J., Banfield J.F. (2014). Microbes in the neonatal intensive care unit resemble those found in the gut of premature infants. Microbiome.

[7] Young G.R., van der Gast C.J., Smith D.L., Berrington J.E., Embleton N.D., Lanyon C. (2020). Acquisition and Development of the Extremely Preterm Infant Microbiota Across Multiple Anatomical Sites. J. Pediatr. Gastroenterol. Nutr..

[8] Azad M.B., Konya T., Maughan H., Guttman D.S., Field C.J., Chari R.S., Sears M.R., Becker A.B., Scott J.A., Kozyrskyj A.L. (2013). Gut microbiota of healthy Canadian infants: Profiles by mode of delivery and infant diet at 4 months. CMAJ.

[9] Stewart C.J., Ajami N.J., O’Brien J.L., Hutchinson D.S., Smith D.P., Wong M.C., Ross M.C., Lloyd R.E., Doddapaneni H., Metcalf G.A. (2018). Temporal development of the gut microbiome in early childhood from the TEDDY study. Nature.

[10] Martin R., Makino H., Cetinyurek Yavuz A., Ben-Amor K., Roelofs M., Ishikawa E., Kubota H., Swinkels S., Sakai T., Oishi K. (2016). Early-Life Events, Including Mode of Delivery and Type of Feeding, Siblings and Gender, Shape the Developing Gut Microbiota. PLoS ONE.

[11] Sevelsted A., Stokholm J., Bonnelykke K., Bisgaard H. (2015). Cesarean section and chronic immune disorders. Pediatrics.

[12] Mayer-Davis E.J., Rifas-Shiman S.L., Zhou L., Hu F.B., Colditz G.A., Gillman M.W. (2006). Breast-feeding and risk for childhood obesity: Does maternal diabetes or obesity status matter?. Diabetes Care.

[13] Ihekweazu F.D., Versalovic J. (2018). Development of the Pediatric Gut Microbiome: Impact on Health and Disease. Am. J. Med. Sci..

[14] Milani C., Duranti S., Bottacini F., Casey E., Turroni F., Mahony J., Belzer C., Delgado Palacio S., Arboleya Montes S., Mancabelli L. (2017). The First Microbial Colonizers of the Human Gut: Composition, Activities, and Health Implications of the Infant Gut Microbiota. Microbiol. Mol. Biol. Rev..

[15] Arrieta M.C., Stiemsma L.T., Dimitriu P.A., Thorson L., Russell S., Yurist-Doutsch S., Kuzeljevic B., Gold M.J., Britton H.M., Lefebvre D.L. (2015). Early infancy microbial and metabolic alterations affect risk of childhood asthma. Sci. Transl. Med..

[16] Stiemsma L.T., Turvey S.E. (2017). Asthma and the microbiome: Defining the critical window in early life. Allergy Asthma Clin. Immunol..

[17] Boutin R.C.T., Sbihi H., McLaughlin R.J., Hahn A.S., Konwar K.M., Loo R.S., Dai D., Petersen C., Brinkman F.S.L., Winsor G.L. (2021). Composition and Associations of the Infant Gut Fungal Microbiota with Environmental Factors and Childhood Allergic Outcomes. mBio.

[18] Duffy L.C. (2000). Interactions mediating bacterial translocation in the immature intestine. J. Nutr..

[19] Halpern M.D., Denning P.W. (2015). The role of intestinal epithelial barrier function in the development of NEC. Tissue Barriers.

[20] Tarr P.I., Warner B.B. (2016). Gut bacteria and late-onset neonatal bloodstream infections in preterm infants. Semin. Fetal Neonatal Med..

[21] Carl M.A., Ndao I.M., Springman A.C., Manning S.D., Johnson J.R., Johnston B.D., Burnham C.A., Weinstock E.S., Weinstock G.M., Wylie T.N. (2014). Sepsis from the gut: The enteric habitat of bacteria that cause late-onset neonatal bloodstream infections. Clin. Infect. Dis..

[22] Sherman M.P. (2010). New concepts of microbial translocation in the neonatal intestine: Mechanisms and prevention. Clin. Perinatol..

[23] Jacquot A., Neveu D., Aujoulat F., Mercier G., Marchandin H., Jumas-Bilak E., Picaud J.C. (2011). Dynamics and clinical evolution of bacterial gut microflora in extremely premature patients. J. Pediatr..

[24] Arboleya S., Binetti A., Salazar N., Fernandez N., Solis G., Hernandez-Barranco A., Margolles A., de Los Reyes-Gavilan C.G., Gueimonde M. (2012). Establishment and development of intestinal microbiota in preterm neonates. FEMS Microbiol. Ecol..

[25] Butel M.J., Suau A., Campeotto F., Magne F., Aires J., Ferraris L., Kalach N., Leroux B., Dupont C. (2007). Conditions of bifidobacterial colonization in preterm infants: A prospective analysis. J. Pediatr. Gastroenterol. Nutr..

[26] Madan J.C., Salari R.C., Saxena D., Davidson L., O’Toole G.A., Moore J.H., Sogin M.L., Foster J.A., Edwards W.H., Palumbo P. (2012). Gut microbial colonisation in premature neonates predicts neonatal sepsis. Arch. Dis. Child. Fetal Neonatal Ed..

[27] Mai V., Torrazza R.M., Ukhanova M., Wang X., Sun Y., Li N., Shuster J., Sharma R., Hudak M.L., Neu J. (2013). Distortions in development of intestinal microbiota associated with late onset sepsis in preterm infants. PLoS ONE.

[28] Zhou Y., Shan G., Sodergren E., Weinstock G., Walker W.A., Gregory K.E. (2015). Longitudinal analysis of the premature infant intestinal microbiome prior to necrotizing enterocolitis: A case-control study. PLoS ONE.

[29] Saiman L., Ludington E., Pfaller M., Rangel-Frausto S., Wiblin R.T., Dawson J., Blumberg H.M., Patterson J.E., Rinaldi M., Edwards J.E. (2000). Risk factors for candidemia in Neonatal Intensive Care Unit patients. The National Epidemiology of Mycosis Survey study group. Pediatr. Infect. Dis. J..

[30] Qin J., Li R., Raes J., Arumugam M., Burgdorf K.S., Manichanh C., Nielsen T., Pons N., Levenez F., Yamada T. (2010). A human gut microbial gene catalogue established by metagenomic sequencing. Nature.

[31] Stoll B.J., Hansen N., Fanaroff A.A., Wright L.L., Carlo W.A., Ehrenkranz R.A., Lemons J.A., Donovan E.F., Stark A.R., Tyson J.E. (2002). Late-onset sepsis in very low birth weight neonates: The experience of the NICHD Neonatal Research Network. Pediatrics.

[32] Russell C., Lay K.M. (1973). Natural history of Candida species and yeasts in the oral cavities of infants. Arch. Oral Biol..

[33] James S.A., Phillips S., Telatin A., Baker D., Ansorge R., Clarke P., Hall L.J., Carding S.R. (2020). Preterm Infants Harbour a Rapidly Changing Mycobiota That Includes *Candida* Pathobionts. J. Fungi.

[34] Manzoni P., Stolfi I., Pugni L., Decembrino L., Magnani C., Vetrano G., Tridapalli E., Corona G., Giovannozzi C., Farina D. (2007). A multicenter, randomized trial of prophylactic fluconazole in preterm neonates. N. Engl. J. Med..

[35] Gunsalus K.T., Tornberg-Belanger S.N., Matthan N.R., Lichtenstein A.H., Kumamoto C.A. (2016). Manipulation of Host Diet to Reduce Gastrointestinal Colonization by the Opportunistic Pathogen *Candida albicans*. mSphere.

[36] Arsenault A.B., Gunsalus K.T.W., Laforce-Nesbitt S.S., Przystac L., DeAngelis E.J., Hurley M.E., Vorel E.S., Tucker R., Matthan N.R., Lichtenstein A.H. (2019). Dietary Supplementation with Medium-Chain Triglycerides Reduces *Candida* Gastrointestinal Colonization in Preterm Infants. Pediatr. Infect. Dis. J..

[37] Bolyen E., Rideout J.R., Dillon M.R., Bokulich N.A., Abnet C.C., Al-Ghalith G.A., Alexander H., Alm E.J., Arumugam M., Asnicar F. (2019). Reproducible, interactive, scalable and extensible microbiome data science using QIIME 2. Nat. Biotechnol..

[38] Callahan B.J., McMurdie P.J., Rosen M.J., Han A.W., Johnson A.J., Holmes S.P. (2016). DADA2: High-resolution sample inference from Illumina amplicon data. Nat. Methods.

[39] Katoh K., Misawa K., Kuma K., Miyata T. (2002). MAFFT: A novel method for rapid multiple sequence alignment based on fast Fourier transform. Nucleic Acids Res..

[40] Price M.N., Dehal P.S., Arkin A.P. (2010). FastTree 2--approximately maximum-likelihood trees for large alignments. PLoS ONE.

[41] Lozupone C.A., Hamady M., Kelley S.T., Knight R. (2007). Quantitative and qualitative beta diversity measures lead to different insights into factors that structure microbial communities. Appl. Environ. Microbiol..

[42] McDonald D., Price M.N., Goodrich J., Nawrocki E.P., DeSantis T.Z., Probst A., Andersen G.L., Knight R., Hugenholtz P. (2012). An improved Greengenes taxonomy with explicit ranks for ecological and evolutionary analyses of bacteria and archaea. ISME J..

[43] Friedman J., Alm E.J. (2012). Inferring correlation networks from genomic survey data. PLoS Comput. Biol..

[44] Koenig J.E., Spor A., Scalfone N., Fricker A.D., Stombaugh J., Knight R., Angenent L.T., Ley R.E. (2011). Succession of microbial consortia in the developing infant gut microbiome. Proc. Natl. Acad. Sci. USA.

[45] La Rosa P.S., Warner B.B., Zhou Y., Weinstock G.M., Sodergren E., Hall-Moore C.M., Stevens H.J., Bennett W.E., Shaikh N., Linneman L.A. (2014). Patterned progression of bacterial populations in the premature infant gut. Proc. Natl. Acad. Sci. USA.

[46] Tauchi H., Yahagi K., Yamauchi T., Hara T., Yamaoka R., Tsukuda N., Watanabe Y., Tajima S., Ochi F., Iwata H. (2019). Gut microbiota development of preterm infants hospitalised in intensive care units. Benef. Microbes.

[47] Schwiertz A., Gruhl B., Lobnitz M., Michel P., Radke M., Blaut M. (2003). Development of the intestinal bacterial composition in hospitalized preterm infants in comparison with breast-fed, full-term infants. Pediatr. Res..

[48] Arboleya S., Ang L., Margolles A., Yiyuan L., Dongya Z., Liang X., Solis G., Fernandez N., de Los Reyes-Gavilan C.G., Gueimonde M. (2012). Deep 16S rRNA metagenomics and quantitative PCR analyses of the premature infant fecal microbiota. Anaerobe.

[49] Fehr K., Moossavi S., Sbihi H., Boutin R.C.T., Bode L., Robertson B., Yonemitsu C., Field C.J., Becker A.B., Mandhane P.J. (2020). Breastmilk Feeding Practices Are Associated with the Co-Occurrence of Bacteria in Mothers’ Milk and the Infant Gut: The CHILD Cohort Study. Cell Host Microbe.

[50] Azad M.B. (2019). Infant Feeding and the Developmental Origins of Chronic Disease in the CHILD Cohort: Role of Human Milk Bioactives and Gut Microbiota. Breastfeed. Med..

[51] Pannaraj P.S., Li F., Cerini C., Bender J.M., Yang S., Rollie A., Adisetiyo H., Zabih S., Lincez P.J., Bittinger K. (2017). Association Between Breast Milk Bacterial Communities and Establishment and Development of the Infant Gut Microbiome. JAMA Pediatr..

