# Supplementary material for: Minimal Effects of Medium-Chain Triglyceride Supplementation on the Intestinal Microbiome Composition of Premature Infants: A Single-Center Pilot Study
Source: Nutrients. 2022 May 22;14(10):2159. doi: 10.3390/nu14102159 (PMC9145469; doi:10.3390/nu14102159)
Supplement: Supplementary file 1 [file nutrients-14-02159-s001.zip › nutrients-1710647-supplementary.pdf]

Supplemental Figures

Supplemental Figure S1

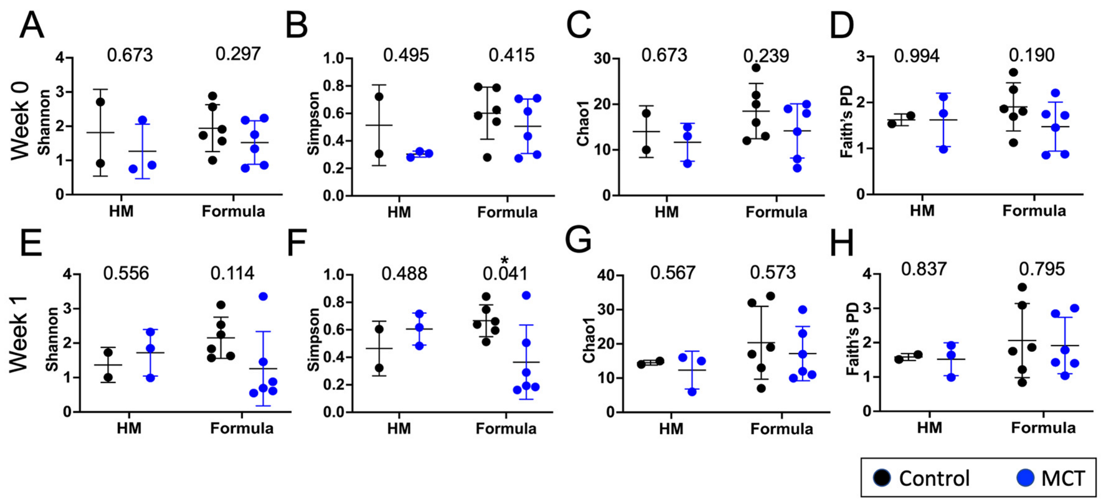

Supplemental Figure S1. Infant gut microbiota diversity is moderately impacted by MCT in infants fed a formula diet. Bacterial composition of control and MCT supplemented infants over three weeks was analyzed using QIIME2 and diversity scores were calculated. Infants were grouped based on diet (human milk (HM) vs formula diet). Bar represents the mean diversity score for all infants within a group. (A) Chao1, (B) Simpson, (C) Shannon, and (D) Faith's PD are diversity scores for infants before MCT supplementation began (week 0). (E) Chao1, (F) Simpson, (G) Shannon, and (H) Faith's PD are diversity scores for infants one week after MCT supplementation (week 1). Black circles denote control infants. Blue circles denote MCT supplemented infants. Mann-

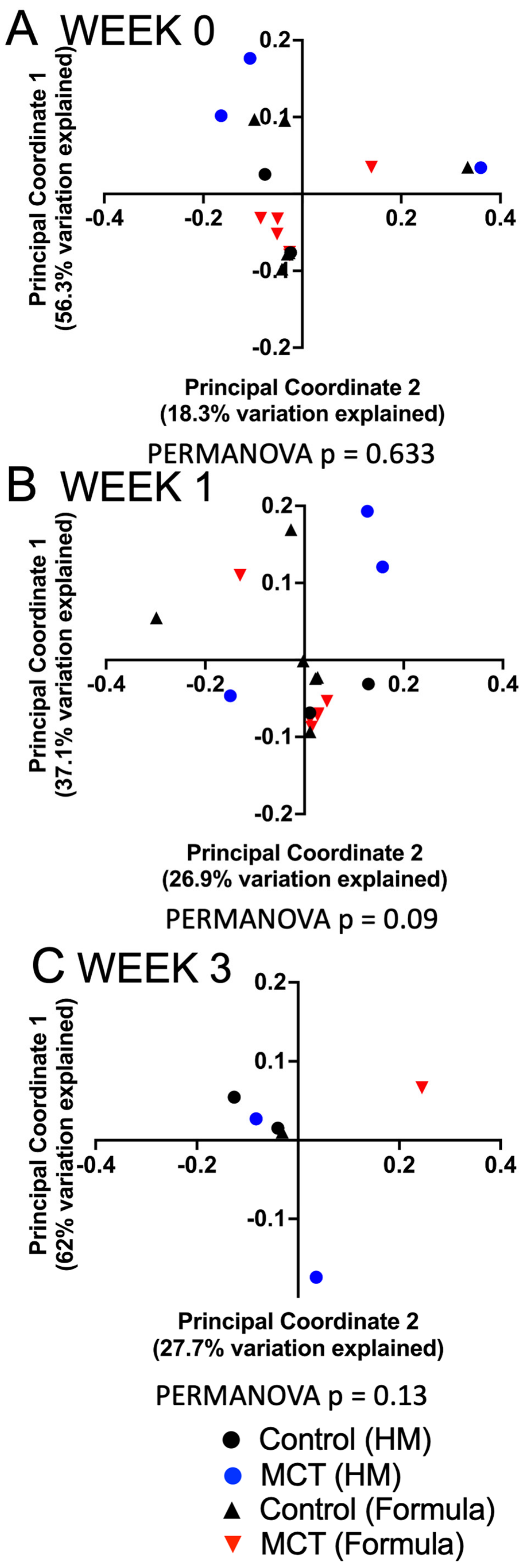

Supplemental Figure S2

Supplemental Figure S2. Analyses of gastrointestinal bacterial communities in control or MCT supplemented infants who received a human milk or formula diet. Bacterial composition of control and MCT supplemented infants over three weeks was analyzed using QIIME 2. Weighted UniFrac distances were used to perform a Principal Coordinate Analysis between groups at different time points and fed different diets. (A) Week 0 indicates samples collected before supplementation began. (B) Week 1 indicates samples collected after one week of MCT supplementation. (C) Week 3 indicates samples collected after three weeks of MCT supplementation. Black circles denote control infants fed human milk. Blue circles denote MCT supplemented infants fed human milk. Black triangles denote control infants given a formula diet. Red triangles denote control infants given a formula diet. PERMANOVA p-values are displayed below each graph.

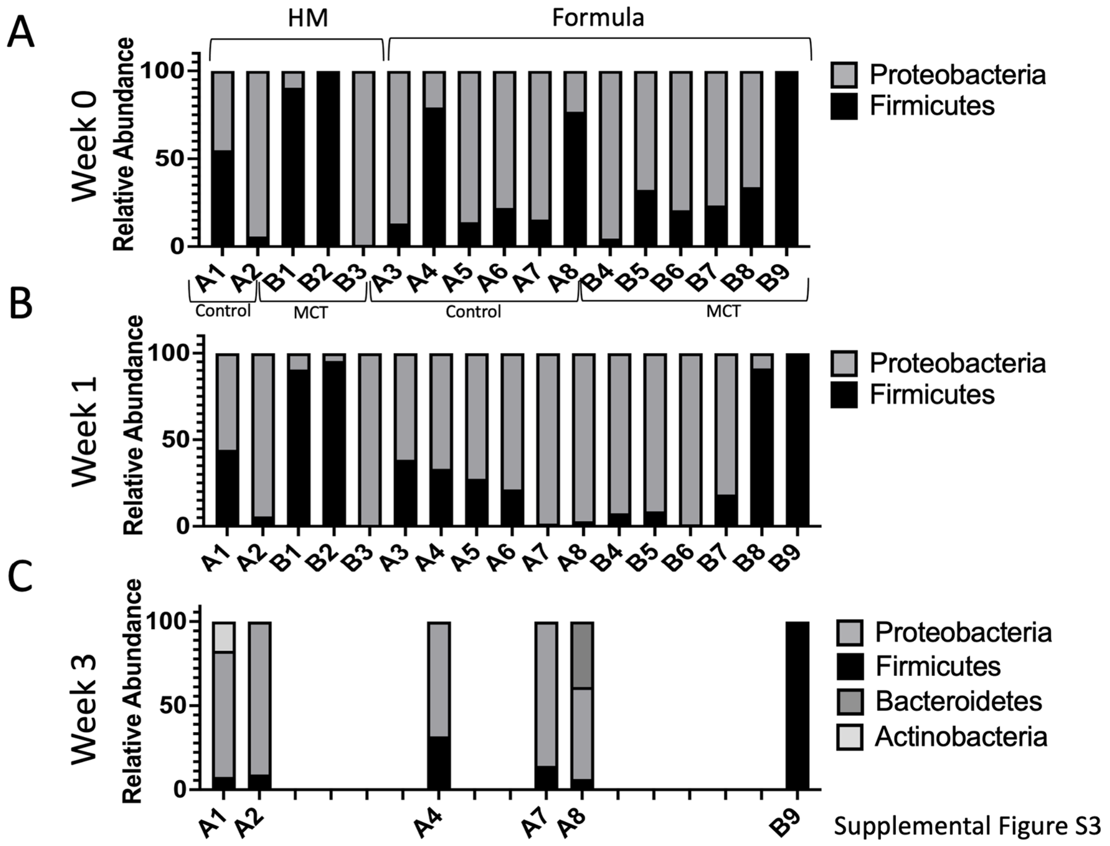

Supplemental Figure S3. Infant gut bacterial phyla from infant stool, grouped by diet. Phyla displayed over three weeks. (A) Week 0 indicates samples collected before supplementation began. (B) Week 1 indicates samples collected after one week of MCT supplementation. (C) Week 3 indicates samples collected after three weeks of MCT supplementation. Infants A1-B3 belong to the human milk group. Infants A3-B9 belong to the formula group. Only a total of six infants remained in the study by week 3.
